# Biomarker Evaluation and Toxic Effects of an Acute Oral and Systemic Fumonisin Exposure of Pigs with a Special Focus on Dietary Fumonisin Esterase Supplementation

**DOI:** 10.3390/toxins10070296

**Published:** 2018-07-17

**Authors:** Hanna Schertz, Sven Dänicke, Jana Frahm, Dian Schatzmayr, Ilse Dohnal, Gerlinde Bichl, Heidi E. Schwartz-Zimmermann, Sonia Colicchia, Gerhard Breves, Jens P. Teifke, Jeannette Kluess

**Affiliations:** 1 Friedrich-Loeffler-Institute, Federal Research Institute for Animal Health, 38116 Braunschweig, Germany; haschertz@gmail.com (H.S.); jana.frahm@fli.de (J.F.); jeannette.kluess@fli.de (J.K.); 2 BIOMIN Holding GmbH, BIOMIN Research Center, 3430 Tulln, Austria; dian.schatzmayr@biomin.net (D.S.); ilse.dohnal@biomin.net (I.D.); gerlinde.bichl@biomin.net (G.B.); 3 Christian Doppler Laboratory for Mycotoxin Metabolism and Center for Analytical Chemistry, IFA, 3430 Tulln, Austria; heidi.schwartz@boku.ac.at (H.E.S.-Z.); sonia.colicchia@romerlabs.com (S.C.); 4 Institute for Physiology, University of Veterinary Medicine Hannover, Foundation, 30559 Hannover, Germany; gerhard.breves@tiho-hannover.de; 5 Friedrich-Loeffler-Institute, Federal Research Institute for Animal Health, Südufer 10, 17493 Greifswald-Insel Riems, Germany; jens.teifke@fli.de

**Keywords:** fumonisin, pigs, Sa/So ratio, single-dose, clinical examination, blood count, clinical biochemistry, fumonisin esterase

## Abstract

The mycotoxin fumonisin B1 (FB1) is a frequent contaminant of feed. It causes a disruption of sphingolipid metabolism and pulmonary, hepatic, and immunological lesions in pigs depending on the exposure scenario. One sensitive biomarker for FB1 exposure is the sphinganine (Sa) to sphingosine (So) ratio in blood. The fumonisin esterase FumD, which can be used as a feed additive, converts FB1 into the much less toxic metabolite hydrolyzed FB1 (HFB1). We conducted a single-dose study with barrows allocated to one of five treatments: (1) control (feed, 0.9% NaCl intravenously *iv*), (2) 139 nmol FB1 or (3) HFB1/kg BW *iv*, (4) 3425 nmol FB1/kg BW orally (*po*), or (5) 3321 nmol FB1/kg BW and 240 U FumD/kg feed *po*. The Sa/So ratio of *iv* and *po* FB1 administered groups was significantly elevated in blood and *Liquor cerebrospinalis*, but no fumonisin-associated differences were reflected in other endpoints. Neither clinical lung affections nor histopathological pulmonary lesions were detected in either group, while some parameters of hematology and clinical biochemistry showed a treatment–time interaction. FumD application resulted in Sa/So ratios comparable to the control, indicating that the enzymatic treatment was effectively preventing the fumonisin-induced disruption of sphingolipid metabolism.

## 1. Introduction

It was reported by the Food and Agriculture Organization (FAO) that approximately 25% of the world’s agricultural commodities are contaminated with mycotoxins, leading to significant economic losses [1]. *Fusarium verticillioides* (=*F. moniliforme*) is one of the main contaminants of animal feed worldwide [2]. In 1988, after years of studying the fungus, the compound responsible for its toxic effects was finally isolated and named fumonisin [2]. Different fumonisins have been reported so far and were grouped into main categories A, B, C, and P. The most abundant among them is fumonisin B_1_ (FB_1_), but co-occurrence with other fumonisins (FB_2_, FB_3_) as well as other mycotoxins such as aflatoxin or zearalenone is possible [3]. 

It is described in the literature that fumonisin toxicity is based on a competitive inhibition of sphinganine (sphingosine) *N*-acyltransferase (ceramide synthase, CerS), a key enzyme in sphingolipid metabolism (Figure S1) [4]. Sphingolipids represent a large class of lipids, characterized by a backbone of sphingoid bases (e.g., sphingosine) and are integral parts of biological membranes involved, e.g., in cell communication and signal transduction. In principal, all fumonisins consist of a long hydroxylated hydrocarbon chain linked to tricarballylic acid, methyl, and amino groups [5] and show thus structural similarity to the original substrates of CerS, sphinganine (Sa), and sphingosine (So). This structural similarity mediates the competitive inhibition of CerS by fumonisins, resulting in a disruption of the sphingolipid metabolism [6,7]. The inhibition of CerS causes an elevation of the sphinganine-to-sphingosine ratio (Sa/So ratio) in tissues and fluids, which can be used as a biomarker for FB_1_ exposure [8,9]. Moreover, differences in CerS expression patterns seem to play a role in inter-species differences of target organs in fumonisin toxicosis [4,7,10].

Consumption of *Fusarium*-contaminated food or feed may lead to teratogenic, cancerogenic, neurotoxic, and immune suppressive effects particularly in animals targeting various organs depending on the exposure scenario. In humans, fumonisins are alleged risk factors for esophageal and liver cancers, neural tube defects, and cardiovascular problems as indicated from mainly rodent models [5,11]. However, so far none of the epidemiological studies on human cohorts could conclusively prove a causal relationship between dietary fumonisin exposure and those diseases [12], although recent studies report good correlations between fumonisin content in complementary, maize-based food and growth stunting in young children [13,14,15]. In pigs, FB_1_ has been shown to be cardio- and hepatotoxic [16] and to cause pulmonary edema (porcine pulmonary edema, PPE) whereby the effects of FB_1_ are time and dose dependent [17]. In horses, fumonisins are neurotoxic, causing equine leukoencephalomalacia (ELEM, [18]). Cattle and poultry are reportedly less sensitive to fumonisins than other species [19]. Due to these adverse health effects, the EFSA panel on contaminants in the food chain reviewed available data on fumonisins in various species and published the following conclusions: in pigs and horses, the lowest observed adverse effect level (LOAEL) of FB_1_ was identified as 0.2 mg/kg body weight (BW)*d, defined as the lowest trigger of fumonisin-related changes in Sa/So ratio of pigs or neuronal aberrations in horses [20]. Lung lesions typical for PPE and their clinical representation are reported to be mainly linked to a hydrothorax and alterations in the heart and circulatory system [17]. In contrast, LOAEL in adult ruminants and poultry chicks are much higher, set at 2.4 mg/kg BW*d and 2.0 mg/kg BW, respectively. In the last decade, many studies were conducted examining chronic fumonisin exposure [6,8,21,22], but still only little is known about acute fumonisin intoxication in pigs [17,23], in particular with respect to its impact on animal health and biomarker development. 

Due to the detrimental effects of mycotoxins, several strategies have been developed for the prevention of fungal growth and the detoxification of mycotoxins in food and feed [1]. One decontamination strategy employs the use of a carboxylesterase (FumD) as feed additive for the transformation of FB_1_ to its biologically inactive metabolite, hydrolyzed FB_1_ (HFB_1_), in the gastrointestinal tract as proven under chronic exposure conditions [24]. However, acute exposure scenarios might occur with abrupt changes between different feed batches. Thus, the aim of the present experiment was to examine the effects of an acute oral fumonisin exposure, either in the absence or presence of FumD, on indicators of fumonisin toxicity such as respiratory impairment, hematology, clinical biochemistry, as well as fumonisin biomarkers. Besides the commonly used biomarker Sa/So-ratio we also intended to investigate their phosphates as they’ve been also recently proposed as potential biomarkers [25]. This study was conducted in the frame of an investigation on toxicokinetics and metabolism of fumonisins and thus included also intravenous application of FB_1_ and its fully-hydrolyzed form, HFB_1_. The two latter substances functioned as references for a complete (100%) systemic bioavailability in the experimental setup along the oral fumonisin administration, considering the generally poor bioavailability of fumonisins in pigs [23,26]. 

## 2. Results

### 2.1. Sphingoid Bases and Their Ratio in Blood and Cerebrospinal Fluid 

**Figure 1:**
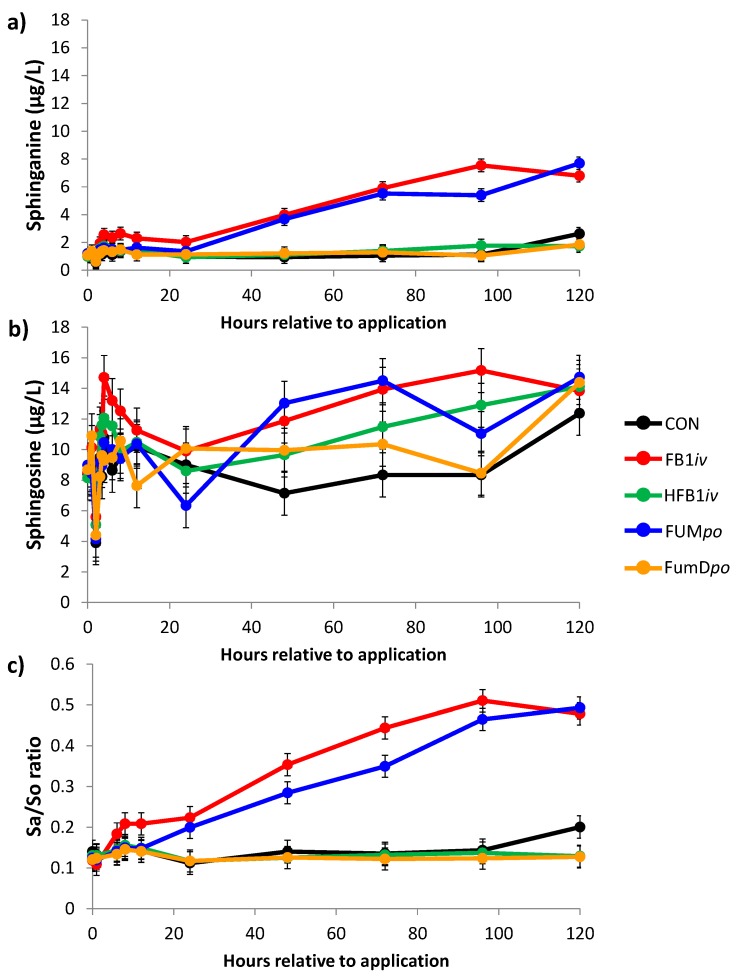
Serum concentration of (**a**) sphinganine (Sa), (**b**) sphingosine (So), and the respective calculated Sa/So ratio (**c**) during the course of the experimental period until 120 h post-toxin application. Data represent LSmeans (±SEM; *n* = 6/group) and statistical main effects were distributed as follows: (**a**) Sa: *p*_group_ < 0.001, *p*_time_ < 0.001, *p*_group × time_ < 0.001; (**b**) So: *p*_group_ = 0.018, *p*_time_ < 0.001, *p*_group × time_ = 0.166; (**c**) Sa/So ratio: *p*_group_ < 0.001, *p*_time_ < 0.001, *p*_group × time_ < 0.001. CON: control; FB1*iv*: 139 nmol FB_1_/kg·BW^−1^; HFB1*iv*: 139 nmol HFB_1_/kg·BW^−1^; FUM*po*: 120 mg FB_1_ + 48 mg FB_2_ + 14 mg FB_3_/kg diet; FumD*po*: 120 mg FB_1_ + 48 mg FB_2_ + 14 mg FB_3_/kg diet and 240 U fumonisin esterase/kg diet).

Serum levels of sphinganine (Sa) and sphingosine (So) were measured in a time dependent manner and their ratio was calculated, serving as a biomarker for fumonisin exposure in pigs (Figure 1). Groups CON and HFB1*iv* showed consistently low and unaltered Sa/So ratios throughout the entire experimental period, whereas for FB1*iv* and FUM*po* an increase in their ratios was observed, which proved to be significantly higher after 24 h. For both FB_1_-exposed groups the Sa/So ratio was still elevated and did not decline until the end of the experiment. The rise in Sa/So ratio was primarily caused by a time-dependent increase in sphinganine concentration upon FB_1_-exposure, whereas sphingosine changes were only of minor importance. 

Levels of sphingoid bases and their ratio detected in the enzyme-treated pigs of group FumD*po*, were comparable to those of CON and HFB1*iv* groups, with no alteration during the course of experiment. The contrasting time kinetics of sphinganine and Sa/So ratio between fumonisin groups FB1*iv* and FUM*po* on one hand and groups CON, HFB1*iv*, and FumD*po* on the other hand were the reason for the highly significant interaction between group and time. 

**Figure 2:**
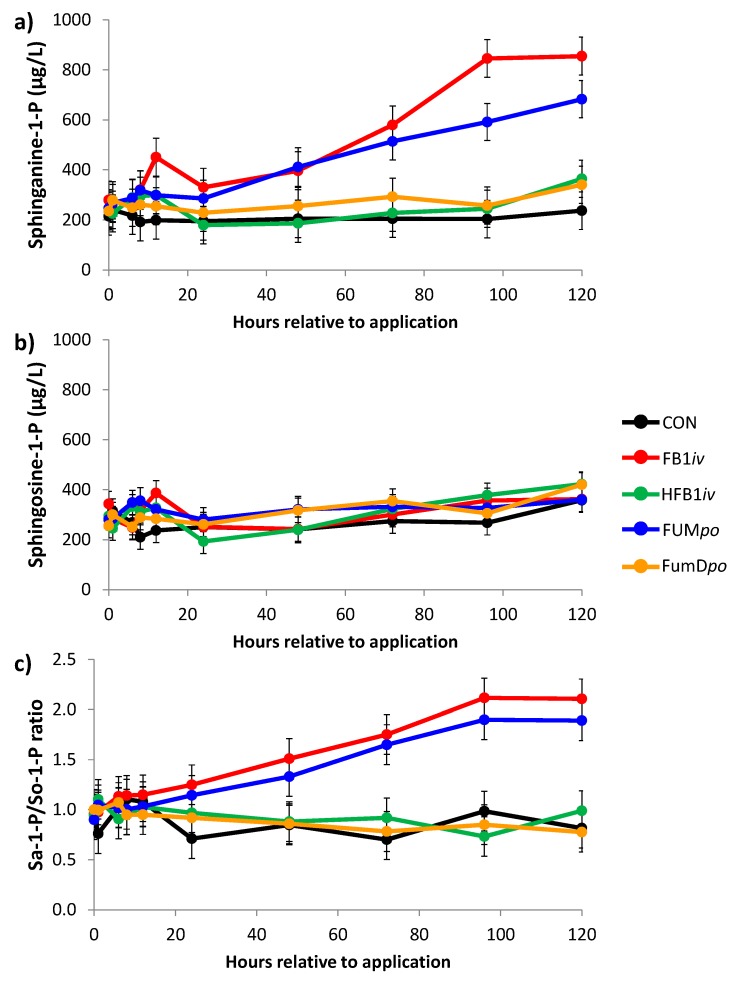
Concentration of (**a**) sphinganine-1-phosphate (Sa-1-P), (**b**) sphingosine-1-phosphate (So-1-P), and the respective calculated Sa-1-P/So-1-P ratio (**c**) in whole blood during the course of the experimental period until 120 h post-toxin application. Data represent LSmeans (±SEM; *n* = 6/group) and statistical main effects were distributed as follows: (**a**) Sa-1-P: *p*_group_ < 0.01, *p*_time_ < 0.001, *p*_group × time_ = 0.043; (**b**) So-1-P: *p*_group_ = 0.542, *p*_time_ < 0.01, *p*_group × time_ = 0.833; (**c**) Sa/So ratio: *p*_group_ < 0.001, *p*_time_ < 0.001, *p*_group × time_ < 0.001. CON: control; FB1*iv*: 139 nmol FB_1_/kg·BW^−1^; HFB1*iv*: 139 nmol HFB_1_/kg·BW^−1^; FUM*po*: 120 mg FB_1_ + 48 mg FB_2_ + 14 mg FB_3_/kg diet; FumD*po*: 120 mg FB_1_ + 48 mg FB_2_ + 14 mg FB_3_/kg diet and 240 U fumonisin esterase/kg diet).

Levels of sphinganine-1-phosphate (Sa-1-P) and sphingosine-1-phosphate (So-1-P) in whole blood were also measured in a time dependent manner and their ratio was calculated (Figure 2). The response to FB_1_-exposure, either *iv* or *po*, was comparable to that of the precursors sphinganine and sphingosin: a significant increase in Sa-1-P and therefore in the Sa-1-P/So-1-P ratio with no alteration of So-1-P except for a time effect. However, it is noteworthy that actual phosphate levels were multiple times higher than Sa (Sa-1-P ~180 fold) and So (So-1-P ~30 fold) and thus the resulting biomarker Sa-1-P/So-1-P ratio was also approximately 4-fold higher. Again, treatment with FumD*po* was capable of preventing any alterations in sphingoid base phosphate levels.

**Figure 3:**
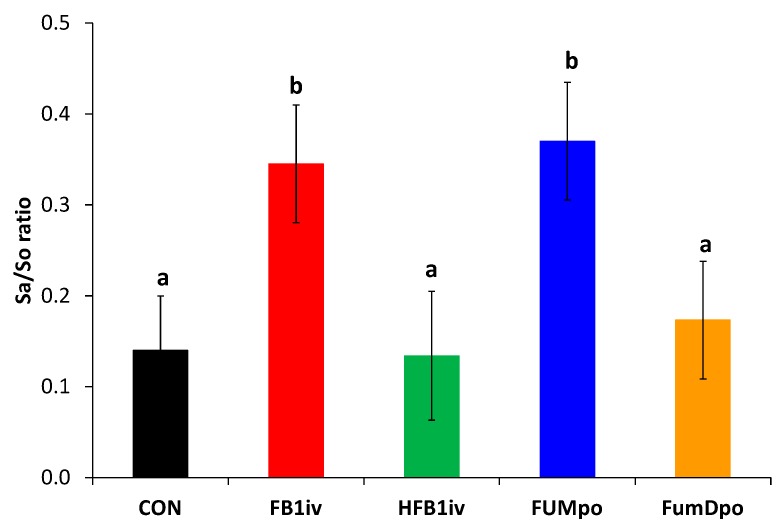
Calculated Sa/So ratio in cerebrospinal fluid at the end of the experimental period (120 h). Data represent LSmeans (±SEM) and statistical main effect was p_group_ = 0.031. Columns with unlike superscripts are significantly different from each other (*post hoc* Student’s *t*-test, *p* < 0.05). CON: control; FB1*iv*: 139 nmol FB_1_/kg·BW^−1^; HFB1*iv*: 139 nmol HFB_1_/kg·BW^−1^; FUM*po*: 120 mg FB_1_ + 48 mg FB_2_ + 14 mg FB_3_/kg diet; FumD*po*: 120 mg FB_1_ + 48 mg FB_2_ + 14 mg FB_3_/kg diet and 240 U fumonisin esterase/kg diet). Columns with unlike superscripts (a, b) are significantly different from each other.

Additionally, Sa and So were measured in *Liquor cerebrospinalis* at the end of the experiment at 120 h and their respective ratio was calculated (Figure 3). Similar ratios to those in blood were determined for each of the treatments, with significantly elevated ratios in groups FB1*iv* and FUM*po* compared to CON, HFB1*iv*, and FumD*po*. 

### 2.2. Clinical Examination and Physiology

#### 2.2.1. Respiratory System

**Figure 4:**
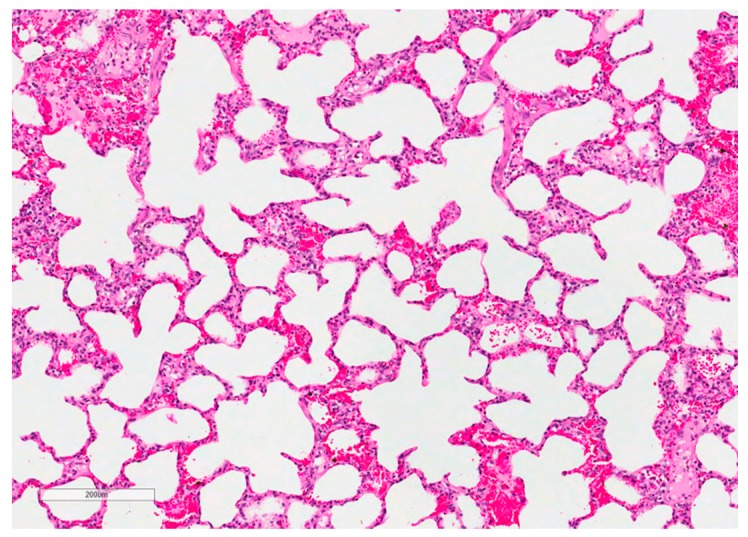
Example of HE-stained, FFPE lung tissue used for quantification of tissue and airway percentage (pig 22, FUM*po*). No marked histological lesions are present.

**Figure 5:**
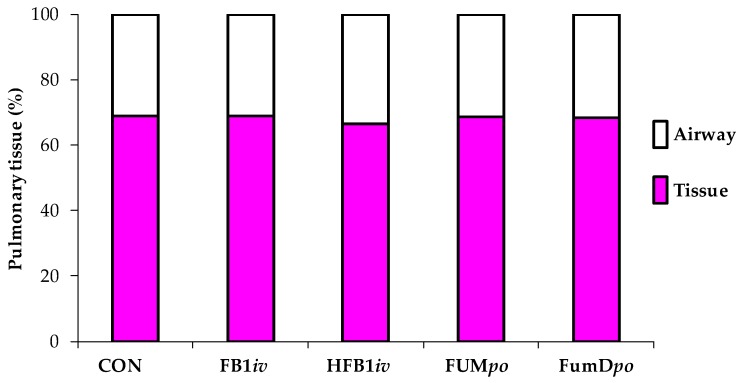
Analysis of lung tissue using Halo^TM^ image analysis software (Indica Labs, Inc., Corrales, NM, USA, 2015), determining tissue and airway proportion of the histological specimen. Data represent LSmeans (*n* = 6/group) and statistical main effects were distributed as *p*_Airway_ = 0.926 and *p*_Tissue_ = 0.926. CON: control; FB1*iv*: 139 nmol FB_1_/kg·BW^−1^; HFB1*iv*: 139 nmol HFB_1_/kg·BW^−1^; FUM*po*: 120 mg FB_1_ + 48 mg FB_2_ + 14 mg FB_3_/kg diet; FumD*po*: 120 mg FB_1_ + 48 mg FB_2_ + 14 mg FB_3_/kg diet and 240 U fumonisin esterase/kg diet).

At slaughter, neither macroscopic alterations in the lungs nor differences in the relative lung weight (LSmeans: CON = 9.78 g/kg BW, FB1*iv* = 9.66 g/kg BW, HFB1*iv* = 10.03 g/kg BW, FUM*po* = 10.61 g/kg BW, FumD*po* = 10.87 g/kg BW) were detected due to treatment (*p*_group_ = 0.909). Lung tissue from both lungs was sampled after slaughter at 120 h post toxin application, formalin-fixed, and HE-stained for further analysis (Figure 4). Specialized software (Halo^TM^ image analysis system) was used to determine the proportion of tissue and airways in each histological specimen (Figure 5): lung tissue comprised ~68% and airway ~32% of the total lung area measured. Statistical evaluation showed no significant differences between treatments for either tissue or airway proportion. There was no indication of edema fluid present in airways in fumonisin-treated animals.

#### 2.2.2. Clinical Examination 

All animals appeared clinically inconspicuous during the entire experimental period. Respiratory rate, heart rate, and body temperature were evaluated in time and results are shown in Table 1. Respiratory rate was influenced by time, whereby the rate increased significantly in pigs of all groups from base levels (t = 0) until 96 h after treatment and then decreased again at 120 h. However, respiratory rate at 120h was still significantly higher than base levels (*post hoc* Student’s *t*-test: base level at t = 0 vs. 24, 48, 72, 96, and 120 h, *p* < 0.01). Moreover, respiratory rate tended to be also affected by treatment (*p* = 0.053) due to the slightly higher respiratory rate in group FB1*iv*. Heart rate displayed no significant main effects or interactions. Body temperature displayed only a significant time effect, slightly increasing from the beginning to the end of the experiment. All clinical parameters were within their respective physiological ranges.

The cumulative clinical score (CCS) represented the entity of nine scored symptoms such as consciousness, respiratory (besides respiratory rate) and cardiovascular system likely to be induced by the administered experimental treatments. There was no statistical difference between treatments (*p* = 0.903) and the very low mean CCS of 6 also indicated no alteration of animal health as such. In comparison, a maximum CCS of 130 would have been potentially achieved in animals with most pronounced clinical symptoms in each category. 

#### 2.2.3. Red Blood Cell Count (RBCC)

In RBCC, significant time effects (*p*_time_ < 0.05) were detected in all parameters with the exception of PDW, but fluctuations within groups were all within their respective physiological range (Table S1). No effect of treatment alone was apparent between the groups, but a significant group–time interaction was detected for MCH, MCHC, and PCT (*p*_group×time_ < 0.05). However, this was also in the respective physiological range.

#### 2.2.4. White Blood Cell Count (WBCC)

Total leukocyte counts (Table S2) varied also significantly over time (*p*_time_ < 0.001), increasing 6 h after treatment in all groups, then decreasing and elevating again at the end of the experiment. Furthermore, statistical analysis revealed a significant time–group interaction for all leukocyte subtypes such as lymphocytes, neutrophils (Figure S2), monocytes, eosinophils, and basophils (Table S2). Although RBCC parameters were statistically significant, changes were within the physiological ranges.

#### 2.2.5. Clinical Biochemistry

Albumin, alkaline phosphatase, γ-glutamyl-transferase, urea, and triglycerides revealed only a significant time effect, whereas for bilirubin and aspartate-amino-transferase, no significant differences were observed (Table 2). Treatment did not influence these parameters, but for albumin there was a tendency for a group–time interaction. This might be attributed to lower albumin values at 24 h in group FB1*iv* (LSmeans: 31.9 vs. 36.8 g/L) and at 96 h for FUM*po* (LSmeans: 33.3 vs. 37.7 g/L), whereas for all other times and treatments no changes were detectable.

**Figure 6:**
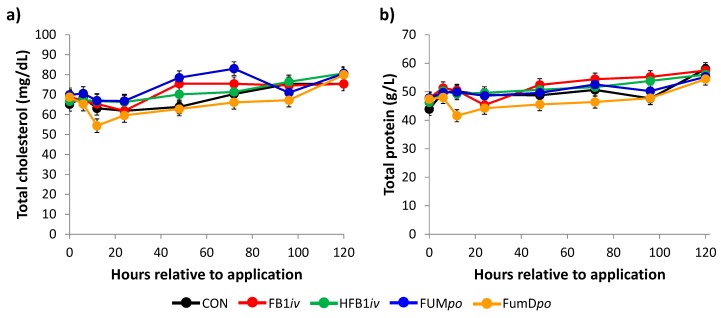
Development of serum (**a**) total cholesterol and (**b**) total protein in pigs allocated to one of five treatments. Data represent LSmeans (±SEM, n = 6/group) and statistical main effects were distributed as follows: (**a**) *p*_group_ = 0.039, *p*_time_ < 0.001, *p*_group×time_ = 0.060 (**b**) *p*_group_ = 0.027, *p*_time_ < 0.001, *p*_group×time_ = 0.345. Reference values for total cholesterol: 77–128 mg/dL (Kraft and Dürr [27]) and total protein: 49.6–72.4 g/L (Kixmöller [28]) for German Landrace pigs. CON: control; FB1*iv*: 139 nmol FB_1_/kg·BW^−1^; HFB1*iv*: 139 nmol HFB_1_/kg·BW^−1^; FUM*po*: 120 mg FB_1_ + 48 mg FB_2_ + 14 mg FB_3_/kg diet; FumD*po*: 120 mg FB_1_ + 48 mg FB_2_ + 14 mg FB_3_/kg diet and 240 U fumonisin esterase/kg diet).

Significant differences for group and time were obtained for total cholesterol (Figure 6a) and a marked trend for an interaction of group–time was also observed. The impact of group was primarily reflected in elevated values for FUM*po* compared to the other groups (CON: 67.94^a^, FB1*iv*: 70.48^abc^, HFB1*iv*: 70.68^ab^, FUM*po*: 73.40^b^, FumD*po*: 65.50^c^ mg/dL; *post hoc* Student’s *t*-test *p* < 0.05), whereby the most pronounced difference was apparent to the enzyme-treated group FumD*po* (*post hoc* Student’s *t*-test, *p* < 0.001). The tendency for an interaction could be mainly attributed to the divergent time kinetics of FUM*po* and FumD*po*: the former indicated a slight increase in cholesterol from 12 to 72 h, whereas the latter evinced an early drop at 12 h and a slow return to near base levels until 120 h. Group FB1*iv* numerically displayed a similar pattern to FUM*po*, but less pronounced. 

In total protein (Figure 6b), similar significant effects were found for group and time, whereas no significant interaction was detectable. The impact of group was mainly due to a lower protein level in group FumD*po* (CON: 49.72^ab^, FB1*iv*: 51.79^a^, HFB1*iv*: 50.95^a^, FUM*po*: 50.45^a^, FumD*po*: 46.93^b^ g/L; *post hoc* Student’s *t*-test *p* < 0.05). Over the total experimental time there was an increase in serum protein in all groups (pooled LSmeans 0 vs. 120 h: 46.63 vs. 56.24 g/L).

## 3. Discussion

The objective of our trial was to investigate the effect of an oral single-dose fumonisin exposure on animal health with special emphasis on the respiratory tract and the biomarker Sa/So ratio, in the absence or presence of the fumonisin esterase FumD. This study was conducted in the frame of an investigation on toxicokinetics and metabolism of fumonisins and thus [26] intravenous applications of FB_1_ and HFB_1_ were also employed.

As shown in previous studies [29], FB_1_ disrupts sphingolipid metabolism and causes an increase in the Sa/So ratio. In our study, the Sa/So ratio in serum was significantly elevated 24 h post dosing in both FB_1_ administered groups—*po* and *iv* application—and this elevation continued to the end of the experimental period at 120 h. We detected no statistical differences in the temporal sequence of the biomarker after *po* or *iv* fumonisin application. Dilkin et al. [17] reported the first significant increase in blood Sa/So ratio after 6 h and the peak Sa/So ratio at 12 h post treatment after a single-dose administration of FB_1_. This ratio was still elevated 96 h after FB_1_ administration and a decline was not observed. Similarly, in our study, we also detected an increased Sa/So ratio until the end of the experiment (120 h) without an indication for a decline. The earlier increase in the Sa/So ratio reported by Dilkin et al. [17] compared to our study might be explained by the 2-fold higher FB_1_ dosage administered by Dilkin and co-workers (5 mg vs. 2.4 mg FB_1_/kg BW in our study), supporting the reported dose-dependency of the biomarker described by others [29]. Another contributing factor in the earlier rise of the biomarker was likely the toxin application in fasted pigs via oral gavage in contrast to our study where fumonisin was administered on top of the morning feed. It can be assumed that toxin absorption in fasted animals is much faster accomplished compared to fed animals, resulting in an earlier response of the sphingoid bases. Riley and colleagues [29] reported a good relationship between animal’s exposure to dietary fumonisins (FB_1_ + FB_2_) and Sa/So ratio in blood as higher fumonisin concentrations and longer exposure both resulted in a near-linear increase of the biomarker. The authors reported a significant increase in serum Sa/So ratio for 5 mg FB_1_ + FB_2_/kg diet (ranging approximately from ~0.29 to 0.49 mg/kg BW) after 14 days and for 39 mg/kg diet (ranging approximately from ~2.7 to 5.2 mg/kg BW) after 5 days of exposure [29]. In this assumption, we estimated a daily weight gain of 300 g per animal, whereby the latter dosage corresponded approximately to the toxin exposure in our own study and also elicited a comparable serum Sa/So ratio. Although there were no earlier times analyzed in the cited study one could assume an earlier rise of the ratio than 5 days. In a study with weaning piglets chronically fed 2 mg FB_1_ + FB_2_/ kg diet with or without 60 U FumD/kg diet for 42 days it was shown that FumD degraded FB_1_ to HFB_1_ in the gastrointestinal tract [8]. Accordingly, in our study, the Sa/So ratio in serum of the FumD*po* group was not significantly different from the control indicating detoxification of FB_1_ by FumD. This detoxification was evident in a reduced bioavailability of FB_1_ and a shift in urine and feces towards HFB_1_, confirming the predictability of the Sa/So ratio as a valid biomarker [26]. 

In cerebrospinal fluid we detected significantly higher Sa/So ratios 120 h post dosage in FB1*iv* and FUM*po* groups compared to CON, HFB1*iv*, and FumD*po* groups, comparable to the ratio calculated in blood samples. Such an increase in Sa/So ratio was reported in the forebrain of 2-day old rat pups [30] injected subcutaneously a single dose of either 0.8 or 8 mg FB_1_/kg BW. This dose-dependent elevation was already detected after 3 h, continued rising until 24 h, and was primarily caused by an increase in sphinganine levels. FB_1_ concentrations showed an inverse development to the Sa/So ratio in brain and plasma, decreasing continuously already from 3 h post-injection. The detection of FB_1_ in brain tissues, albeit in very low concentration, was also reported in growing pigs fed 45 mg FB_1_/kg diet for 10 days [31] or treated with a single-dose application of ^14^C-labelled FB_1_ intravenously or intragastrically [23]. However, in growing mice, this increase of sphinganine in the brain after fumonisin exposure was less evident [32]: mice were injected either 10 or 100 µg FB_1_/animal subcutaneously for 7d and only in cortex of high-dose mice a significant sphinganine rise was shown, whereas the variation in the low-dose group was too high for any statistical effect. All other regions (midbrain, cerebellum, medulla oblongata) showed no change in sphinganine at all. Only FB_1_ infusion directly into the lateral ventricle of the brain showed for both dosages significant increases in sphinganine levels in most brain regions analyzed. This illustrates that on one hand age or developmental stage of the animal and on the other hand exposure scenario of FB_1_ are crucial in this context. 

We suggest the following possible explanations for an elevated Sa/So ratio in cerebrospinal fluid in our study: (**1**) FB_1_ could cross the blood brain barrier (BBB), interfering with or depleting the myelin sheath and thereby elevating the Sa/So ratio in liquor. The studies cited above support this notion, but the FB_1_ level in *Liquor cerebrospinalis* was below the limit of detection (LOD < 1 ng/mL) in our study after 120 h. However, this might indicate that already trace amounts of fumonisin crossing the BBB can yield a reaction, i.e., a rise in Sa/So ratio in the central nervous system or that there’s a considerable time lag between relevant levels of FB_1_ crossing the BBB and the reaction in sphingoid bases. As we could not obtain any cerebrospinal fluid before 120 h, we cannot answer this question conclusively. (**2**) FB_1_ might elicit a second messenger response in attaching to BBB-structures on the blood side, mediating a biomarker increase in *Liquor cerebrospinalis*. (**3**) Both sphingoid bases present in the circulatory system might simply cross the BBB and thus increase the Sa/So ratio in *Liquor cerebrospinalis*, because neutral, lipophilic substances with low molecular weight such as sphingoid bases diffuse easily over cell membranes [33]. In our study, a significant positive correlation (r = 0.4, *p* = 0.032) between Sa/So ratio in blood and cerebrospinal fluid 120 h after toxin application was calculated and might support this assumption. 

The inhibition of ceramide synthase (CerS) as proposed mode-of-action of FB_1_ in animals [10] would affect two pathways of sphingolipid metabolism: the ceramide de novo synthesis with the enzymatic conversion of sphinganine (=dihydrosphingosine) to dihydroceramide and the salvage pathway, in which sphingosine, available from sphingolipid degradation, is re-acylated to ceramide [34]. The blockage of the de novo synthesis would result in higher levels of sphinganine, which would not be further converted into ceramide. This is supported by our data on blood sphingoid bases with a rise in sphinganine and hardly any change in sphingosine. Furthermore, the downstream conversion of both bases into their phosphates Sa-1-P and So-1-P via sphingosine kinases corresponds to the development of their precursors, i.e., increased Sa-1-P and no changes in So-1-P in blood. An inhibition of CerS in the salvage pathway should result in an increase of sphingosine as it would not be acylated into ceramide.

The second option (attachment FB_1_ on BBB, eliciting second messenger responses) can be neither proven nor rejected based on data generated in our experiment. However, instead of FB_1_ itself, it is conceivable that sphingosine/sphinganine-1-P (S-1-P) might act on the BBB (among other tissues). S-1-P was already reported to elicit a G-protein coupled second messenger response in various tissues such as the vascular, nervous, or immune system [35,36].

The species-specific clinical symptoms, in particular the affinity to the respiratory tract in pigs in contrast to the central nervous system in horses, is still in need of clarification. In our experiment, pigs were mainly clinically inapparent throughout the entire trial period and the histology of lung tissues showed no differences in airway to tissue proportion, a first indicator for potential lung edema, in response to fumonisin exposure, either. However, besides significant time effects in respiratory rate and body temperature of the animals, irrespective of treatment and most likely due to the excitement initiated by the experimental handling, the respiratory rate tended to be higher in the FB1*iv* group compared to the FUM*po* group. Whether this finding is indeed a reflection of the low oral bioavailability of 3.1% [26] and of an acute toxic effect due to a 100% fumonisin bioavailability after *iv* administration cannot conclusively be answered. Fodor and co-workers [37], who administered similar fumonisin dosages to those we used, detected no clinical alterations, either. In contrast, Dilkin and colleagues [17] reported clinical alterations such as lethargy, ruffled coat, increased heart and respiratory rate, reduced water and feed intake, and preferred lateral recumbency from day two onwards after a single-dose exposure. One explanation for this difference in results could be the administration of double the dose in the Dilkin trial compared to our experiment. This dose-response issue is also highlighted in chronic exposure studies: Colvin et al. [38] dosed male pigs (n = 3) with 32 mg FB_1_/kg BW*day via oral gavage and pigs developed severe clinical signs such as feed refusal and pulmonary edema within 3 days. Riley et al. [29] reported also a clear dose-response of fumonisin toxicity with hepatic injury characterized by significantly elevated biochemical parameters (AST, ALT, γ-GT, ALP) and histological damage (hepatocyte cord disorganization, single cell necrosis, inflammation) starting from 101 mg to 175 mg FB1 + FB2/kg feed*d (corresponding to ~6.7 and 11.7 mg FB1 + FB2/kg BW*d), although liver histopathology already showed tissue damage in some pigs at 39 mg FB1 + FB2/kg feed*d. Respiratory distress appeared only at the highest fumonisin exposure with 175 mg FB_1_ + FB_2_/kg feed*d starting at day 4 to 7 after onset of exposure and was confirmed histologically as severe pulmonary interstitial edema. As in our study, we estimated similar doses it can be assumed that time of exposure makes a difference in damage just as dosage level makes a difference in target organs. This was also confirmed by Motelin et al. [39] who applied fumonisin to male castrated cross-bred pigs (6–13 kg BW) for 14 days. In that study, only pigs fed the highest dose (175 mg/kg feed ~17.5 mg FB1 + FB2/kg BW*d) showed respiratory distress with pulmonary edema and pleural effusion after mycotoxin intake of 4 to 6 days and changes in clinical biochemistry. In the other groups, only histopathological evidence of hepatic injury was present in pigs fed diets ≥23 mg FB1 + FB2/kg BW*d. 

The impact of FB_1_ on clinical-biochemical parameters, indicative for potential damage to organs such as liver and kidneys, was also observed in our study. We detected statistical differences in cholesterol levels between FUM*po* and FumD*po* group with an increased level in the orally treated fumonisin group. An elevation of total blood cholesterol in FUM*po* pigs could be caused by FB_1_-induced liver tissue damage, which was already described in the literature [17,37]. However, in our study, the observed alterations were well within the physiological range, thus the biological relevance in terms of an impaired animal health might be debatable. The effect of fumonisins on cholesterol has been described previously: Dilkin et al. [17] reported a significant increase in cholesterol 96 h after oral dosage of 5 mg FB_1_/kg BW, albeit also in the physiological range. However, in contrast to their observations, in our experiment we did not detect any fumonisin-related increases in ALP and AST. Gelderblom and co-workers also described a significant increase of cholesterol after 21 days of feeding 250 mg FB_1_/kg to rats [40] and Rotter and colleagues detected an increase of cholesterol after administering low fumonisin doses (1 mg FB_1_/kg) to swine in the fattening phase until reaching market weight [41]. A recent study on the organ-specific impact of fumonisin [42] demonstrated among others the contrasting effect of fumonisin on ceramide and sphingomyelin levels in liver and lungs, accompanied by an increase in blood cholesterol after 9 days of oral treatment with 1.5 mg FB_1_/kg BW: in liver tissue, d18:1 ceramide levels increased and conversely most sphingomyelin species decreased, whereas the situation was opposite in lungs. Both compounds are usually transported in high-density lipoproteins (HDL) in blood [43] and should therefore be a part of the total cholesterol measurement. Thus, it is conceivable that the commonly detected increase in blood cholesterol in fumonisin-exposed pigs, such as in group FUM*po* in our trial, is due to the change in ceramides and sphingomyelins in liver and lungs being exported from the cells into the blood circulation. 

As discussed above, fumonisins are known to affect the sphingolipid metabolism whereby cell membranes and cellular function might be affected which might also be relevant for blood cells. Although for both red (RBCC) and white blood cell counts (WBCC), significant time effects as well as interactions were observed in our study, these were all in their respective physiological range and without a directed impact of actual treatment. Literature findings also suggest no relevant effect of single-dose fumonisin administration on white blood counts. Dilkin et al. [17] reported no red and white blood cell count alterations after single oral dose of 5 mg FB_1_/kg BW (culture material) in pigs. Likewise, Prelusky et al. who conducted a study administering single doses of 0.5 mg ^14^C-labelled FB_1_/kg BW per oral and 0.4 mg ^14^C-labelled FB_1_/kg BW *iv* to pigs also failed to detect alterations in red or white blood cells counts.

In summary, in our experimental setup with a single-dose fumonisin exposure, the biomarker Sa/So ratio was significantly elevated from 24 h onwards, irrespective of route of administration (*po* or *iv*), and remained elevated in serum until the end of the experiment at 120 h. This rise in ratio was solely due to a marked elevation of sphinganine. Also, their phosphates (Sa-1-P, So-1-P, ratio) showed the same development, pointing towards the inhibition of de novo ceramide synthesis. Although the Sa/So ratio in cerebrospinal fluid at 120 h was also raised in fumonisin-treated pigs, the underlying mechanisms still require further elucidation. With the applied dose, no clinical symptoms and no histological alterations in lung tissue were detected. The administration of fumonisin esterase FumD as feed additive prevented an elevation of the biomarker, supporting our data on the fumonisin conversion into its fully-hydrolyzed metabolite. 

## 4. Materials and Methods 

The experiment was conducted according to the European Community regulations concerning the protection of experimental animals and the guidelines of the German Animal Welfare Act and was approved by the Lower Saxony State Office for Consumer Protection and Food Safety (33.92-42502-04-13/1153, date of approval: 11 July 2013).

### 4.1. Animals, Housing, and Diets

**Figure 7:**
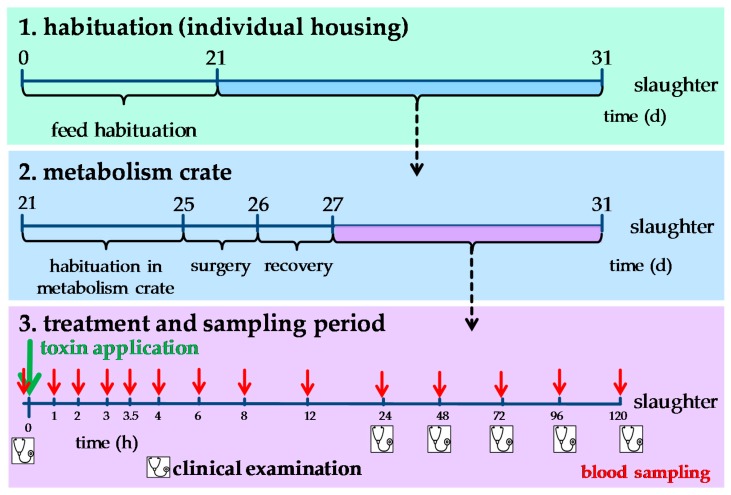
Experimental Design. The trial lasted for 31 days in total. On day 25, pigs were surgically equipped with indwelling venous catheters (left and right jugular vein), followed by a recovery day. During morning feeding on day 27, animals were exposed to oral or intravenous single-dose treatment. Blood samples, indicated by red arrows in panel 3, were taken over a period of 120 h for analyses of biochemical parameters, red and white blood cell count and Sa/So analysis. Moreover, clinical examinations (indicated by stethoscope) were performed on day 27 before toxin application and then every 24 h (6 times in total).

The study was accomplished using 31 barrows (10 weeks old at the start of experiment; German Landrace, Mariensee, Germany), housed individually and fed 700 g of a barley-based diet two times daily for the entire duration of this experiment, formulated to meet or exceed requirements according to GfE recommendations (Table 3). Pigs were kept individually in floor pens for 21 days and thereafter moved to metabolism crates facilitating blood sampling in the subsequent experimental period. After four days of adaption in metabolism crates (body weight [BW]: 34.4 kg ± 2.7 kg), pigs were fasted overnight and surgically equipped with indwelling venous catheters (Silastic^®^, Medical Grade Tubing. 1.57 mm ID × 3.18 mm OD, Dow Corning, Midland, MI, USA) in both *Venae jugulares externae* as previously described [44]. Then, animals had one day for recovery until treatments and sampling period started (Figure 7). Catheters were flushed regularly with heparinized saline solution (2 mL heparin/500 mL physiological saline) to ensure patency.

### 4.2. Experimental Setup

The experimental setup (Figure 7) was described earlier [20]. Briefly, animals were allocated to one of five single-dose treatments (Table 4) with a subsequent sampling period of 120 h. For both oral treatments (FUM*po*, FumD*po*), culture material of *Fusarium verticillioides* (Romer Labs GmbH, Tulln, Austria) and for FumD*po* a fumonisin esterase preparation (FUM*zyme*^®^, BIOMIN, Tulln, Austria) were applied on top of the basal diet. Samples of FUM*po* and FumD*po* morning rations were analyzed by HPLC-MS/MS and contained 80.9 ± 6.9 mg FB_1_, 33.2 ± 3 mg FB_2_, 6.1 ± 1.9 mg FB_3_/kg feed in both contaminated rations (Romer Labs GmbH, Tulln, Austria).

Serial blood samples were collected until 120 h after treatments (Figure 7). Clinical examination of each pig was performed once before implementation of treatments (base level, day 27, Figure 7) and then subsequently every day prior to morning feeding. At 120 h post treatment (day 31), pigs were weighed and then sacrificed by exsanguination following electrical stunning. Organ weights were recorded and fluid (bile, *Liquor cerebrospinalis*) and lung tissue samples collected for analysis. 

### 4.3. Measurements and Analyses

#### 4.3.1. Clinical Examination

Twelve clinical symptoms characterized as possibly associated with fumonisin exposure were examined in a time kinetic manner and nine of them were scored as detailed in Table 5. Clinical scored symptoms were summarized as a cumulative clinical score (CCS) whereby a maximum of 26 points, denoting the worst clinical presentation, were possible at each time. The remaining three symptoms, respiratory rate, heart rate, and body temperature, were evaluated separately as they were measured rather than scored.

#### 4.3.2. Red and White Blood Cell Counts (RBCC, WBCC) and Differential Leukocyte Count

Blood samples (taken immediately before and 6, 12, 24, 48, 72, 96, and 120 h after treatment; EDTA Monovette^®^, Sarstedt AG & Co., Sarstedt, Germany) were analyzed by an automated hematology system (Celltac MEK 6400, Nihon Kohden Europe GmbH, Rosbach, Germany) for RBCC and total leukocyte counts immediately after sampling. Additionally, blood smears were prepared (in duplicate), air-dried, stained according to pappenheim as described earlier [44], and analyzed using bright field microscopy (1000× magnification; Nikon Eclipse E200, Nikon GmbH, Tokyo, Japan) for morphological differentiation of 100 leukocytes per field of vision of each blood smear. 

#### 4.3.3. Clinical Biochemistry

Blood samples (taken immediately before and 6, 12, 24, 48, 72, 96, and 120 h after dosage; Serum Monovette^®^, Sarstedt AG & Co., Sarstedt, Germany; Figure 5) were kept upright at room temperature for at least 30 min for clotting, subsequently centrifuged at 2123× *g* for 15 min, and serum aliquots were stored at −20 °C until analysis. After thawing, following blood biochemical parameters were analyzed using a photometer (Eurolyser CCA 180, Eurolyser Diagnostica GmbH, Salzburg, Austria): total blood cholesterol (CHOL, mg/dL), protein (PROT, g/L), albumine (ALB, g/L), alkaline phosphatase (AP, U/L), total bilirubin (BILI, mg/dL), gamma-glutamyl-transferase (γGT, U/L), aspartate-amino transferase (AST, U/L), urea (UREA, mg/dL), and triacylglycerides (TRI, mg/dL). Reagents used for photometric analysis were obtained from Greiner Diagnostic GmbH (Greiner Diagnostic GmbH, Bahlingen, Germany). 

#### 4.3.4. Sphinganine and Sphingosine Analysis in Serum and *Liquor cerebrospinalis*

Blood samples (taken immediately before and 1, 2, 3, 3.5, 4, 6, 8, 12, 24, 48, 72, 96, and 120 h after dosage; Serum Monovette^®^, Sarstedt AG & Co., Sarstedt, Germany) were kept upright at room temperature for at least 30 min, then centrifuged at 2123× g for 15 min. Serum aliquots and liquor were stored at −20 °C. Sample preparation for determination of sphinganine (Sa) and sphingosine (So) in serum was done according to Grenier, et al. [46] and LC-MS/MS analysis was carried out according to Masching and co-workers [8] on a 1290 Infinity series HPLC system (Agilent Technologies, Waldbronn, Germany) coupled to a Triple Quad 5500 mass spectrometer (SCIEX, Foster City, CA, USA) equipped with a Turbo V electrospray ionization (ESI) source. Chromatographic separation was achieved at 30 °C on a Kinetex C18 column (150 × 2.1 mm i.d., 2.6 μm, Phenomenex, Aschaffenburg, Germany) in gradient elution mode using methanol/water/formic acid (39.85/60/0.15 *v*/*v*/*v*) and methanol/formic acid (99.85/0.15 *v*/*v*) as eluent A and B, respectively. After an initial time of 0.2 min at 35% of B, the proportion of B was increased linearly to 100% within 6.5 min, followed by a hold time of 3.5 min at 100% B. Then, the column was re-equilibrated at 65% A and 35% B for 2.4 min, giving a total run time of 12.5 min. The flow rate was 250 μL/min, the injection volume was 1 μL. MS/MS analysis was performed in the selected reaction monitoring (SRM) mode in positive polarity. The following settings were applied to the ESI source: temperature 550 °C, spray voltage +5500 V, curtain gas 40 psi, ion source gas 1 and ion source gas 2 50 psi. Two transitions were monitored to detect and quantify each compound. 

Sample preparation of *Liquor cerebrospinalis* was similar to serum preparation. Briefly, 200 µL of liquor were precipitated with 0.6 mL of methanol/acetonitrile (50/50, *v*/*v*) and pellets were re-extracted after centrifugation with 0.3 mL of methanol/water (80/20, *v*/*v*). The combined supernatants were dried and the residues were taken up in 600 µL of methanol/water (80/20, *v*/*v*). HPLC-MS/MS analysis was performed as described above for serum. Method validation for pig liquor samples was performed by spiking a pooled liquor sample with appropriate amounts of Sa and So standards prior to extraction in triplicate at six different concentration levels (corresponding to a working range of 0.3–100 ng/mL in measurement solutions for both analytes). The overall recovery of spiked samples was 91 ± 3% RSD (relative standard deviation) for Sa and 94 ± 3% RSD for So. LODs were calculated according to the equation LOD = 3s + m, where s corresponds to the standard deviation and m corresponds to the average of the calculated concentrations of 15 blank runs. Similarly, LOQs were calculated using LOQ = 10s + m. LOD was 0.06 ng/mL liquor and LOQ was 0.2 ng/mL for Sa and So. Repeatability, calculated as RSD of samples spiked in triplicate, was on average 3.7% and 7.5% for Sa and So, respectively.

For the analysis of the sphingoid base-1-phosphates in blood, 50 µL of whole blood was pipetted onto Whatman^®^ protein saver cards (Sigma Aldrich, Vienna, Austria), and the cards were allowed to dry and stored at −20 °C until analysis. For extraction of sphingolipids, the entire blood spots were cut and transferred into 2 mL Eppendorf reaction vials. Subsequently, 2 mL of methanol was added and the analytes were extracted by sonication for 1 h. After removal of the protein saver card discs all samples were centrifuged (14,000× *g* for 10 min). The supernatants were evaporated to dryness, the residues were taken up in 500 μL of methanol, and the solutions were transferred to HPLC vials. 

Detection and quantification of So-1-P and Sa-1-P was performed with an Agilent 1290 UHPLC system (Agilent Technology, Waldbronn, Germany) coupled to a 4000 QTrap mass spectrometer (SCIEX, Foster City, CA, USA) equipped with a Turbo V electrospray ionization (ESI) source. Chromatographic separation was achieved at 30 °C on a Gemini C18 column (150 × 4.6 mm i.d., 5 µm, Phenomenex, Aschaffenburg, Germany) in gradient elution mode using the same eluents as used for serum analysis. After an initial hold time of 0.2 min at 35% B, the proportion of B was increased linearly to 100% within 6.8 min, followed by a hold time of 5.5 min at 100% B. Then the column was re-equilibrated at 65% A and 35% B for 2.4 min. The total run time was 15 min. The flow rate was 900 µL/min, the injection volume 2 µL. The LC stream was directed to MS between 4 and 9.5 min. MS/MS analysis was performed in the selected reaction monitoring (SRM) in positive polarity. The following settings were applied to the ESI source: temperature 550 °C, spray voltage +4200 V, curtain gas 30 psi, ion source gas 1 and ion source gas 2 50 psi. Two transitions were monitored to detect and quantify each compound. For Sa-1-P, the following transitions were chosen: quantifier (quant): *m*/*z* 382.3→284.4 (declustering potential (DP) +86 V, collision energy (CE) +21 eV); qualifier (qual): *m*/*z* 382.3→266.3 (DP +86 V, CE +27 eV). The transitions for So-1-P were: quant: m/z 380.3→264.4 (DP +81 V, CE +24 eV); qual: *m*/*z* 380.3→362.4 (DP +81 V, CE +15 eV). Sa-1-P and So-1-P for calibration were purchased from Avanti Polar Lipids (Alabaster, AL, USA). Sphingoid base-1-phosphats were quantified based on pure standard calibration curves (6 levels, working range 10–300 ng/mL).

#### 4.3.5. Lung Histopathology

**Figure 8:**
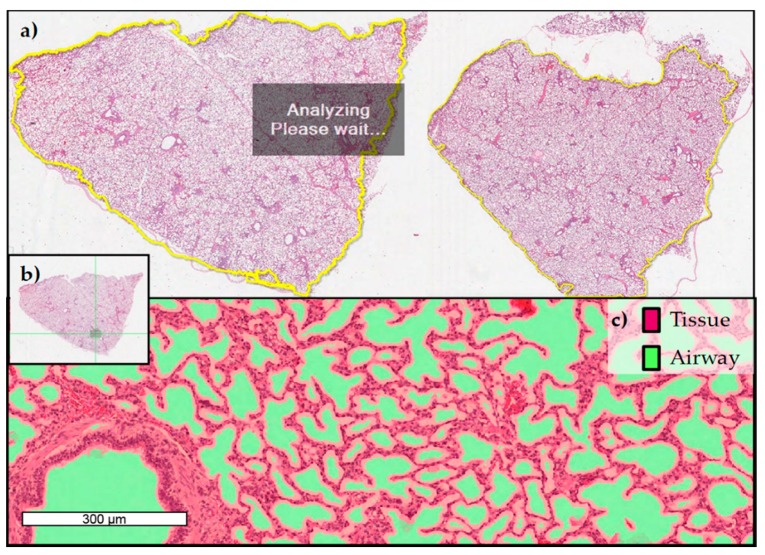
Overview of histopathological analysis of HE-stained, FFPE lung specimen collected at slaughter (120 h after toxin application) in order to evaluate airway and tissue proportion. (**a**) Screenshot of digitized HE-stained lung specimen using image analysis software (HALO^TM^, Indica Labs; Corrales, NM) (**b**) Selected area of quantification within one histological section **(c)** Example from Halo^TM^ Tissue Classifier module (HALO^TM^, Indica Labs; Corrales, NM) with lung tissue marked in red and air-containing areas marked in green.

Pieces of lung tissue (1 × 1 cm wide and 2 cm into the depth) from left and right lung were taken from *Facies parietalis* of the cranial part of *Lobus caudalis* and placed directly into 4% formaldehyde (Roti^®^ HistoFix, Carl Roth GmbH + Co KG, Karlsruhe, Germany) until histological analysis, whereby the fixative was changed once 24 h post sampling. Tissue samples were processed, paraffin-embedded (FFPE-samples) and stained with hematoxylin and eosin (HE). Stained lung tissue sections were digitized at 200× absolute resolution using an Aperio AT2 scanner (Leica Biosystems, Wetzlar, Germany) and analysis of potential atelectasia and interstitial fibrosis on digitized images was performed using an image analysis platform (HALO^TM^, Indica Labs; Corrales, NM, USA). Quantitative analyses of the total lung tissue area (%) vs. air containing areas (%) were obtained with the Halo^TM^ Tissue Classifier module under the supervision of a pathologist. Figure 8 provides an overview of the workflow.

### 4.4. Statistics

All statistics were carried out using SAS software (SAS, version 6.1 for Windows, SAS Institute, Cary, NC, USA). Generally, the procedure “MIXED” was used with group (five experimental groups), time (except for lung histology and liquor analyses), and their interaction as fixed factors with a compound symmetry covariance structure for all analyses as justified by the corrected Akaike information criterion (AICC). For statistical analyses with several time points, base levels at time t = 0 were included as co-variable. LSmeans differences (Student’s *t*-test) were regarded to be significant at a likelihood lower or equal to 0.05 while a tendency or trend was assumed for probabilities lower than 0.1 and higher than 0.5. Values < LOD were estimated as 0.

Due to the non-parametric nature of the clinical score, data were evaluated with a Kruskal–Wallis-procedure (Dell Statistica 13, Dell Inc., Tulsa, OK, USA). Respiratory rate, heart rate, and body temperature were evaluated separately including time as repeated measurements factor, whereby the examination directly before toxin application was set as a co-variable. 

Correlation coefficients between different parameters were estimated using Dell Statistica version 13 software for the Windows™ operating system (Dell Inc., Tulsa, OK, USA).

## Figures and Tables

**Table 1:** Respiratory rate, heart rate, and rectal temperature presented as pooled LSmeans of times (h relative to toxin application) or groups (*n* = 6/group).

Time after Toxin Application (h)	Group	Respiratory Rate (Breaths∙min^−1^)	Heart Rate (Beats∙min^−1^)	Rectal Temperature (°C)
….	CON	33	82	39.3
….	FB1*iv*	39	78	39.2
….	HFB1*iv*	35	75	39.3
….	FUM*po*	32	76	39.2
….	FumD*po*	34	68	39.2
0	….	27	78	38.7
24	….	32	73	39.2
48	….	35	76	39.4
72	….	38	82	39.3
96	….	41	77	39.5
120	….	35	69	39.3
**Main effects, *p*-values (*F*-test)**				
Group		0.053	0.093	0.349
Time		<0.001	0.187	<0.001
Group×Time		0.903	0.979	0.923
PSEM ^§^		±2	±4	±0.1

^§^ pooled standard error of LSmeans.

**Table 2:** Clinical biochemistry.

Time after Toxin Application (h)	ALB (g/L) 19–39 ^†^	ALP (U/L) <170 ^†^	BILI (mg/dL) <0.25 ^†^	γGT (U/L) <45 ^†^	AST (U/L) <35 ^†^	UREA (mg/dL) 20–50 ^†^	TRI (mg/dL) <44 ^†^
0	36.54	127.00	0.16	33.73	n.m. ^‡^	15.47	19.55
6	37.34	113.52	0.15	32.30	24.63	16.94	29.24
12	36.81	108.76	0.17	30.77	21.80	16.95	24.86
24	35.84	105.32	0.16	32.04	22.59	15.18	26.71
48	36.64	102.65	0.16	34.09	22.28	16.91	27.27
72	37.58	109.15	0.16	33.32	22.14	16.90	31.14
96	36.85	108.72	0.16	34.46	25.61	17.14	30.43
120	39.04	107.25	0.16	37.03	26.99	17.51	28.49
**Main effects (*F*-test *p*-value)**							
Group	0.206	0.890	0.740	0.390	0.943	0.355	0.657
Time	<0.001	<0.001	0.399	<0.001	0.600	0.034	<0.001
Group × Time	0.055	0.271	0.631	0.263	0.130	0.919	0.901
PSEM ^§^	±0.50	±4.11	±0.01	±0.92	±2.82	±0.62	±1.58

Data are presented as pooled LSmeans for all groups over time. ALB = albumin; ALP = alkaline phosphatase; BILI = bilirubin; γGT = gamma-glutamyl-transferase; AST = aspartate-amino-transferase; TRI = triglycerides. ^†^ Reference values according to Kraft and Dürr [27]; ^‡^ omitted from statistical evaluation due to technically caused missing values; ^§^ PSEM pooled standard error of means.

**Table 3:** Composition of basal diet.

**Ingredients (g/kg)**	
Barley	745
Soybean meal	190
Soybean oil	25
HCl-lysine	5
DL-methionine	3
L-threonine	2
L-tryptophan	1
Mineral and vitamin premix ^1^	30
**Analyzed composition**	
Dry matter (DM, %)	90.1
Crude protein (g/kg DM)	182.7
Crude fat (g/kg DM)	42.3
Crude fibre (g/kg DM)	43.5
Crude ash (g/kg DM)	65.9
Aflatoxin B_1_, B_2_, G_1_, G_2_ (LOQ < 0.2 μg/g) ^2^	<LOD
Fumonisin B_1_ and B_2_ (LOQ < 20 μg/g) ^2^	<LOD

^1^ provided per kg premix: crude ash 90%, Ca 24.5%, P 6%, Na 5.5%, Mg 1%, Fe 4000 mg, Cu 1000 mg, Mn 2000 mg, Zn 4000 mg, I 50 mg, Se 15 mg, Co 20 mg, vitamin A 400,000 I.U., vitamin D_3_ 40,000 I.U., vitamin E 1200 mg, vitamin B_1_ 37.5 mg, vitamin B_2_ 100 mg, vitamin B_6_ 100 mg, vitamin B_12_ 750 mg, vitamin K_3_ 52.5 mg, nicotinic acid 500 mg, pantothenic acid 337.5 mg, choline chloride 5000 mg. ^2^ analyzed by Romer Labs GmbH.

**Table 4:** Experimental setup.

Group	Fumonisin Application	Dose (nmol·kg·BW^−1^)	Fumonisin Esterase (U/kg Feed)	n
**CON**	0.9% NaCl *iv*	-	-	6 (+1 ^‡^)
**FB1*iv***	100 µg FB_1_/kg BW *iv* ^◊^	139 FB_1_	-	6
**HFB1*iv***	56.2 µg HFB_1_/kg BW *iv* ^◊^	139 HFB_1_	-	6
**FUM*po***	culture material	3377 FB_1_^†^	-	6
	on top of morning ration, *po*	1367 FB_2_^†^		
	(calculated: 120 mg FB_1_ + 48 mg FB_2_ + 14 mg FB_3_/kg diet), 0.9% NaCl *iv*	584 FB_3_^†^		
**FumD*po***	culture material	3321 FB_1_^†^	240	6
	on top of morning ration, *po*	1344 FB_2_^†^		
	(calculated: 120 mg FB_1_ + 48 mg FB_2_ + 14 mg FB_3_/kg diet), 0.9% NaCl *iv*	575 FB_3_^†^		

^◊^ FB_1_ standard provided by Romer Labs GmbH, Tulln, Austria; HFB_1_ standard prepared as described in Hahn, et al. [45]. ^†^ Calculation: based on analysis of culture material (Romer Labs GmbH, Tulln, Austria) and animals’ body weight (BW) at time of application. ^‡^ One pig in CON removed its venous catheter and thus blood sampling was obtained for this individual.

**Table 5:** Cumulative clinical score (CCS) was calculated from all scores of each clinical examination performed over the whole observation period.

Clinical Symptom	Manifestation	Score
Consciousness	unaltered	0
	slightly limited: listless	1
	medium limited: somnolent	2
	highly limited: stupor	3
	comatose	4
Behavior	unaltered	0
	smacking	1
	retching/vomiting	2
	gnashing of teeth	3
Central nervous system	unaltered	0
	nystagmus	1
	shivering	2
	spasms	3
	Grand Mal seizure	4
Coat	unruffled, smooth	0
	ruffled	1
Skin	unmodified	0
	skin alterations	1
	skin surface injured	2
Respiratory difficulties	none	0
	mild labored breathing	1
	medium labored breathing	2
	severe labored breathing	3
	open-mouth breathing	4
Conjunctivae	physiological (pink)	0
	(rose) red	1
	red	2
	cyanosis	3
	anemic	4
Episcleral vessels	physiological	0
	slightly injected	1
	medium injected	2
	highly injected	3
Intestinal symptoms	none	0
	present	1
Maximum CCS per time		26
Maximum CCS (5 times×CCS)		130

## Supplementary Materials

The following are available online at http://www.mdpi.com/2072-6651/10/7/296/s1, Table S1: Red blood cell count (RBCC) for all groups over 120 h sampling period (LSmeans, *n* = 6/group), Table S2: Total (G/L) and differential leukocytes count (% of total leukocyte count) for all groups over a 120 h sampling period (LSmeans, *n* = 6/group), Figure S1: Inhibition of ceramide synthase (CerS) by fumonisins. Adapted and adjusted from Voss, Smith and Haschek [11], Figure S2: (a) Lymphocyte and (b) neutrophil granulocytes proportion over the entire experimental period.
