# Supplementary material for: Biomarker Evaluation and Toxic Effects of an Acute Oral and Systemic Fumonisin Exposure of Pigs with a Special Focus on Dietary Fumonisin Esterase Supplementation
Source: Toxins (Basel). 2018 Jul 17;10(7):296. doi: 10.3390/toxins10070296 (PMC6071024; doi:10.3390/toxins10070296)
Supplement: Supplementary file 1 [file toxins-10-00296-s001.pdf]

Supplementary Materials: Biomarker Evaluation and Toxic Effects of an Acute Oral and Systemic Fumonisin Exposure of Pigs with a Special Focus on Dietary Fumonisin Esterase Supplementation

Hanna Schertz, Sven Dänicke, Jana Frahm, Dian Schatzmayr, Ilse Dohnal, Gerlinde Bichl, Heidi E. Schwartz-Zimmermann, Sonia Colicchia, Gerhard Breves, Jens P. Teifke and Jeannette Kluess

Table S1. Red blood cell count (RBCC) for all groups over 120 h sampling period (LSmeans, n = 6/group).

		Time after toxin application (h)									Main effects, p-values (F-test)		
	Group	0	6	12	24	48	72	96	120	±PSEM§	Group	Time	Group×Time
**RBC**	**CON**	6.44	6.23	5.67	5.79	5.80	5.13	5.18	5.74	0.24	0.800	0.004	0.717
T/L	**FB1*iv***	6.15	5.67	6.89	5.43	6.78	5.63	5.30	5.74	0.24			
(5.8-8.1)*	**HFB1*iv***	6.57	5.51	6.37	5.03	5.14	6.04	5.06	5.53	0.24			
	**FUM*po***	6.42	5.85	5.41	5.72	6.16	5.87	5.31	5.90	0.24			
	**FumD*po***	6.44	5.50	5.20	5.71	5.47	5.30	5.18	5.64	0.24			
**HGB**	**CON**	7.12	6.76	6.14	5.64	6.44	5.66	5.79	6.44	0.21	0.613	0.001	0.636
mmol/L	**FB1*iv***	6.88	6.26	7.44	6.01	7.48	6.33	5.98	6.51	0.21			
(6.7-9.2)*	**HFB1*iv***	7.45	6.29	7.12	5.69	5.74	6.69	5.81	6.32	0.21			
	**FUM*po***	7.11	6.79	6.20	6.23	7.23	6.75	6.16	6.93	0.21			
	**FumD*po***	7.60	6.39	6.06	6.67	5.99	6.20	6.05	6.73	0.21			
**HCT**	**CON**	0.36	0.34	0.31	0.32	0.32	0.29	0.29	0.33	0.01	0.778	0.010	0.739
L/L	**FB1*iv***	0.34	0.31	0.37	0.30	0.37	0.31	0.30	0.33	0.01			
(0.30-	**HFB1*iv***	0.36	0.31	0.35	0.28	0.29	0.34	0.29	0.32	0.01			
0.42)**	**FUM*po***	0.36	0.33	0.31	0.32	0.36	0.33	0.31	0.35	0.01			
	**FumD*po***	0.36	0.31	0.29	0.32	0.31	0.30	0.30	0.33	0.01			
**MCV**	**CON**	56.02	55.69	55.61	56.11	56.37	56.66	57.06	57.69	0.14	0.762	<0.001	0.903
fL	**FB1*iv***	56.02	55.87	55.72	56.14	56.37	56.91	57.52	58.16	0.14			
(50-65)*	**HFB1*iv***	56.02	55.96	55.72	56.31	56.42	56.79	57.49	58.19	0.14			
	**FUM*po***	56.01	56.05	56.16	56.28	56.53	56.75	57.06	58.21	0.14			
	**FumD*po***	56.01	55.65	55.91	56.03	56.36	56.86	57.03	58.13	0.14			
**MCH**	**CON**	1.14	1.13	1.13	1.04	1.15	1.14	1.15	1.16	0.01	0.394	<0.001	<0.001
fmol	**FB1*iv***	1.14	1.13	1.12	1.13	1.13	1.14	1.15	1.15	0.01			
(1.0-1.3)*	**HFB1*iv***	1.14	1.15	1.13	1.14	1.13	1.14	1.15	1.15	0.01			

	**FUM*po***	1.14	1.18	1.16	1.13	1.16	1.17	1.17	1.19	0.01			
	**FumD*po***	1.15	1.13	1.14	1.15	1.09	1.15	1.14	1.16	0.01			
**MCHC**	**CON**	20.25	20.15	20.16	18.39	20.21	19.91	20.02	19.88	0.21	0.503	0.004	0.020
mmol/L	**FB1*iv***	20.35	20.23	20.12	20.12	20.11	20.00	19.94	19.78	0.21			
(19-22)*	**HFB1*iv***	20.35	20.52	20.16	20.22	20.02	20.05	19.99	19.74	0.21			
	**FUM*po***	20.26	20.88	20.59	19.91	20.41	20.49	20.47	20.32	0.21			
	**FumD*po***	20.45	20.34	20.42	20.41	19.24	20.13	19.89	19.97	0.21			
**RDW**	**CON**	16.31	16.32	16.22	16.46	16.31	16.76	16.67	17.07	0.10	0.919	<0.001	0.549
% CV	**FB1*iv***	16.40	16.35	16.57	16.30	16.63	16.43	16.62	17.08	0.10			
(0-50)*	**HFB1*iv***	16.36	16.51	16.32	16.21	16.32	16.62	16.69	17.19	0.10			
	**FUM*po***	16.34	16.17	16.27	16.39	16.22	16.57	16.42	17.09	0.10			
	**FumD*po***	16.35	16.47	16.37	16.27	16.52	16.65	16.58	17.18	0.10			
**PLT**	**CON**	417.26	369.76	327.43	374.76	347.76	438.10	476.26	459.93	18.64	0.215	<0.001	0.051
G/L	**FB1*iv***	438.98	361.48	295.15	358.31	306.31	435.15	404.98	389.15	19.37			
(220-620)*	**HFB1*iv***	406.00	395.66	328.83	392.50	465.16	479.66	547.66	512.50	19.32			
	**FUM*po***	438.12	382.95	340.78	348.45	364.28	412.45	411.28	396.28	19.29			
	**FumD*po***	410.81	352.98	356.48	340.31	418.31	421.81	440.98	430.15	18.94			
**PCT**	**CON**	0.21	0.18	0.16	0.20	0.18	0.22	0.25	0.24	0.01	0.677	<0.001	0.037
%	**FB1*iv***	0.25	0.20	0.14	0.20	0.16	0.25	0.23	0.23	0.01			
	**HFB1*iv***	0.27	0.21	0.16	0.15	0.20	0.25	0.25	0.31	0.01			
	**FUM*po***	0.21	0.21	0.19	0.17	0.19	0.21	0.22	0.22	0.01			
	**FumD*po***	0.22	0.16	0.18	0.17	0.22	0.23	0.23	0.23	0.01			
**MPV**	**CON**	5.37	5.13	5.07	5.72	5.45	5.35	5.60	5.47	0.11	0.889	<0.001	0.153
fL	**FB1*iv***	5.36	5.28	4.96	5.40	5.43	5.45	5.41	5.63	0.11			
	**HFB1*iv***	5.60	5.35	4.98	5.17	5.47	5.63	5.62	5.75	0.11			
	**FUM*po***	5.65	5.37	5.14	5.15	5.47	5.80	5.65	5.49	0.11			
	**FumD*po***	5.37	4.65	5.03	5.33	5.61	5.55	5.38	5.60	0.11			
**PDW**	**CON**	14.71	15.38	15.81	15.28	14.93	14.96	15.08	15.75	0.17	0.528	0.672	0.063
%	**FB1*iv***	14.77	14.66	15.29	15.19	14.97	15.47	14.89	15.11	0.17			
	**HFB1*iv***	14.90	14.95	15.10	14.83	15.67	15.18	14.77	15.03	0.17			
	**FUM*po***	14.82	15.37	15.70	15.32	14.94	15.70	15.04	14.94	0.17			
	**FumD*po***	16.29	14.92	14.92	14.48	14.58	14.42	14.85	14.45	0.18			

Reference values according to *Kraft and Dürr [25] or **Kixmöller [26]; RBC = red blood cells, HGB = hemoglobin, HCT = hematocrit, MCV = mean cell volume, MCH = mean corpuscular hemoglobin, MCHC = mean corpuscular hemoglobin concentration, RDW = red cell distribution width, PLT = platelets/thrombocytes, PCT =

plateletcrit/thrombocrit, MPV = mean platelet volume , PDW = platelet distribution width. Results were evaluated with SAS using t = 0 as co-variable for each parameter. § PSEM pooled standard error of means.

Table S2. Total (G/L) and differential leukocytes count (% of total leukocyte count) for all groups over a 120 h sampling period (LSmeans, *n* = 6/group).

		**Time after toxin application (h)**									**Main effects (*F*-test *p*-value)**		
	**Group**	**0**	**6**	**12**	**24**	**48**	**72**	**96**	**120**	**±PSEM**	**Group**	**Time**	**Group×Time**
**Leukocytes**	**CON**	15.5	18.9	17.1	16.0	16.6	16.2	17.4	20.5	0.8	0.234	<0.001	0.172
G/L	**FB1*iv***	17.4	19.0	19.9	16.1	16.0	17.2	15.5	17.9	0.8			
(10-22)*	**HFB1*iv***	16.2	18.8	15.8	17.6	16.0	19.3	18.4	19.1	0.8			
	**FUM*po***	16.6	19.1	17.6	14.5	15.8	13.9	14.2	16.8	0.8			
	**FumD*po***	15.6	16.4	16.9	14.3	14.9	14.3	15.5	16.7	0.8			
**Monocytes**	**CON**	3.1	5.1	7.8	9.6	6.3	2.8	2.6	3.5	0.3	0.003	0.059	<0.001
%	**FB1*iv***	2.8	4.0	3.3	2.9	3.1	3.9	4.7	3.9	0.3			
(0-5)*	**HFB1*iv***	3.7	2.4	3.1	4.5	4.3	3.6	5.5	5.8	0.3			
	**FUM*po***	3.5	3.5	1.8	3.0	5.8	4.8	5.3	1.7	0.3			
	**FumD*po***	4.6	1.6	2.9	1.9	3.4	2.3	5.1	4.9	0.3			
**Eosinophils**	**CON**	1.4	1.4	5.1	1.9	2.6	1.8	0.1	0.5	0.2	0.696	<0.001	<0.001
%	**FB1*iv***	1.7	4.7	3.8	2.9	1.4	1.1	0.7	1.2	0.2			
(0-6)*	**HFB1*iv***	1.5	2.6	3.5	2.3	1.5	0.7	0.6	1.3	0.2			
	**FUM*po***	1.2	3.2	5.0	1.0	1.2	1.0	0.7	1.9	0.2			
	**FumD*po***	2.1	4.1	4.8	2.3	0.8	1.0	1.5	0.2	0.3			
**Basophils**	**CON**	0.1	0.6	0.1	0.1	0.4	0.5	0.3	0.1	0.1	0.014	0.304	<0.001
%	**FB1*iv***	0.4	0.2	0.4	0.7	0.2	0.5	0.9	0.6	0.1			
(0-2)*	**HFB1*iv***	0.2	0.2	0.0	0.8	0.1	0.4	0.6	0.0	0.1			
	**FUM*po***	0.3	0.0	0.3	0.3	1.0	0.5	0.0	0.2	0.1			
	**FumD*po***	0.6	0.4	0.6	0.4	0.6	0.4	0.7	1.2	0.1			

*Reference values according to Kraft and Dürr [25]. Results were evaluated with SAS using t = 0 as co-variable for each parameter.

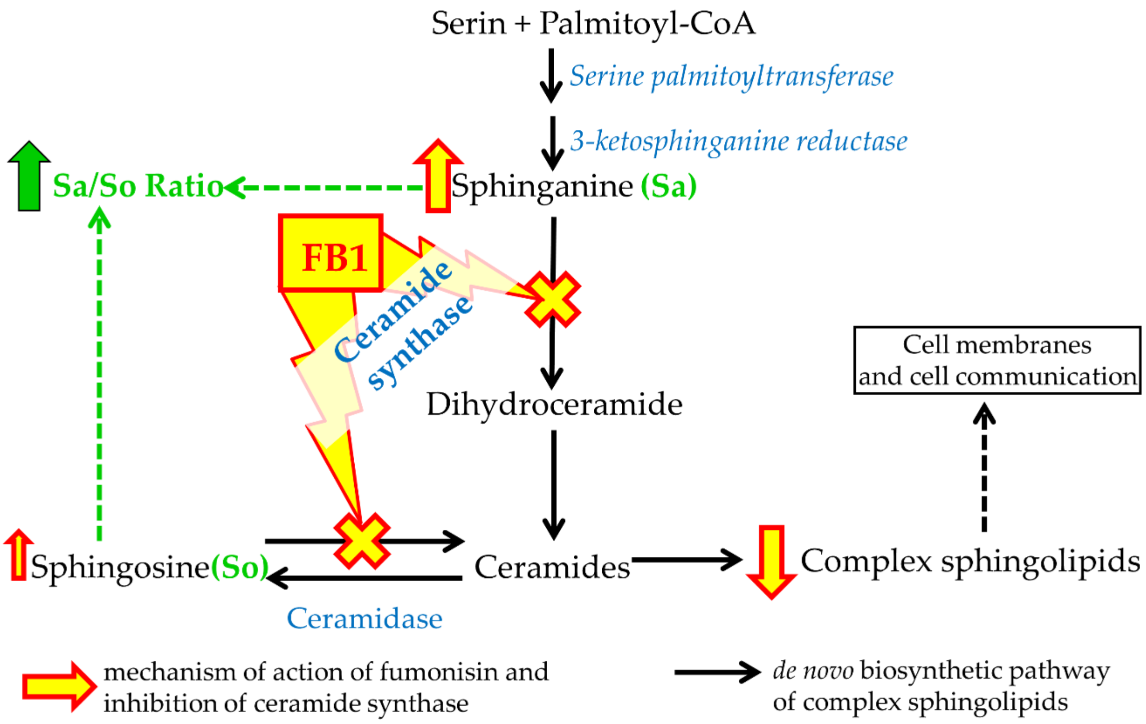

Figure S1. Inhibition of ceramide synthase (CerS) by fumonisins. Adapted and adjusted from Voss, Smith and Haschek [11].

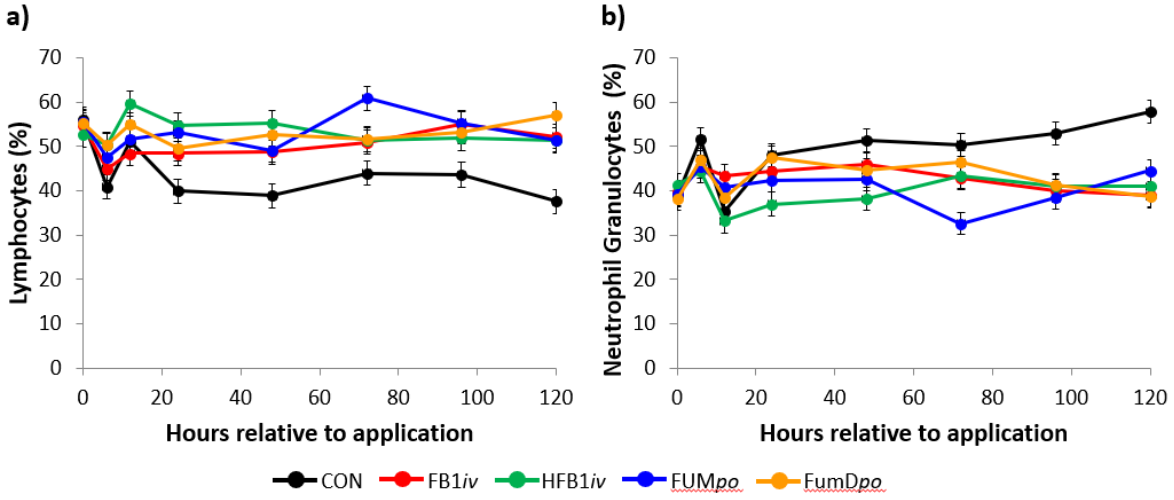

Figure S2. **(a)** Lymphocyte and **(b)** neutrophil granulocytes proportion over the entire experimental period. Data represent LSmeans (± SEM, *n* = 6/group) and statistical main effects were distributed as follows: **(a)** $p_{group} < 0.001$, $p_{time} < 0.001$, $p_{group×time} < 0.001$; **(b)** $p_{group} < 0.001$, $p_{time} < 0.001$, $p_{group×time} < 0.001$. Reference values lymphocytes: 49–85% and neutrophil granulocytes: 10–46% (Kraft and Dürr [25]). CON: control; FB1iv: 139 nmol FB1/kg·BW−1; HFB1iv: 139 nmol HFB1/kg·BW−1; FUMpo: 120 mg FB1 + 48 mg FB2 + 14 mg FB3/kg diet; FumDpo: 120 mg FB1 + 48 mg FB2 + 14 mg FB3/kg diet and 240 U fumonisin esterase/kg diet).
